# Attitudes and Beliefs of Older Black Americans Toward the COVID-19 Virus and Vaccine

**DOI:** 10.1093/geroni/igab046.3209

**Published:** 2021-12-17

**Authors:** Dwana Bass, Sophie Hanna, Sarah Shair, Loraine DiCerbo, Bruno Giordani, Voyko Kavcic

**Affiliations:** 1 Institute of Gerontology - Wayne State University, Sterling Heights, Michigan, United States; 2 Wayne State University, Detroit, Michigan, United States; 3 University of Michigan, University of Michigan, Michigan, United States; 4 Neuropsychology/Michigan Alzheimer's Disease Research Center, University of Michigan, Ann Arbor, Michigan, United States

## Abstract

The COVID-19 pandemic, an unprecedented health emergency, has devastated the nation, and disproportionately affected persons of color, especially Black Americans. It has forced health officials to rapidly develop and distribute COVID-19 vaccines, resulting in the importance of understanding Black Americans’ attitudes and beliefs about COVID-19. We analyzed experiences of 167 Black Americans, ages 65 and older, recruited from Wayne State Institute of Gerontology Healthier Black Elders Center and surrounding communities. Participants were telephoned starting September 2020 and given the GAD-7 anxiety scale and a COVID-19 questionnaire measuring demographics, stressors, and emotional responses associated with the COVID-19 pandemic. A scale was also designed, adapted from the Health Belief Model, to measure fear of getting COVID-19, beliefs about the origins of COVID-19, uncertainty about vaccine safety, and intent to be vaccinated (5-point Likert scale). Of the 167 participants, 112 (67%) said they would agree to vaccination, 24 (14%) were ambivalent, and 31 (19%) said they would decline. T-tests comparing pro- and anti-vaccine participants showed that those not planning to get vaccinated expressed lower generalized anxiety (p=.002), COVID-19 fear (p<.001), and concerns about vaccine safety (p=.01), but greater belief that COVID-19 is man-made (p=.05). The current study provides a snapshot of urban Black American older adults who are in general eager to get vaccinated for COVID-19. Counterintuitively, those unwilling to accept the COVID-19 vaccine also had lower concerns for vaccine safety. More research is needed to fully understand the attitudes and beliefs of this underserved population regarding the COVID-19 virus and vaccine.

